# Opto-thermoelectric microswimmers

**DOI:** 10.1038/s41377-020-00378-5

**Published:** 2020-08-17

**Authors:** Xiaolei Peng, Zhihan Chen, Pavana Siddhartha Kollipara, Yaoran Liu, Jie Fang, Linhan Lin, Yuebing Zheng

**Affiliations:** 1 Materials Science & Engineering Program and Texas Materials Institute, The University of Texas at Austin, Austin, TX 78712 USA; 2 Walker Department of Mechanical Engineering, The University of Texas at Austin, Austin, TX 78712 USA; 3 State Key Laboratory of Precision Measurement Technology and Instruments, Department of Precision Instrument, Tsinghua University, Beijing, 100084 People’s Republic of China

**Keywords:** Optical manipulation and tweezers, Applied optics, Optical physics

## Abstract

Inspired by the “run-and-tumble” behaviours of Escherichia coli (*E. coli*) cells, we develop opto-thermoelectric microswimmers. The microswimmers are based on dielectric-Au Janus particles driven by a self-sustained electrical field that arises from the asymmetric optothermal response of the particles. Upon illumination by a defocused laser beam, the Janus particles exhibit an optically generated temperature gradient along the particle surfaces, leading to an opto-thermoelectrical field that propels the particles. We further discover that the swimming direction is determined by the particle orientation. To enable navigation of the swimmers, we propose a new optomechanical approach to drive the in-plane rotation of Janus particles under a temperature-gradient-induced electrical field using a focused laser beam. Timing the rotation laser beam allows us to position the particles at any desired orientation and thus to actively control the swimming direction with high efficiency. By incorporating dark-field optical imaging and a feedback control algorithm, we achieve automated propelling and navigation of the microswimmers. Our opto-thermoelectric microswimmers could find applications in the study of opto-thermoelectrical coupling in dynamic colloidal systems, active matter, biomedical sensing, and targeted drug delivery.

## Optical actuation: microparticle swimming

Dielectric-metal microstructures that are propelled by light can swim and navigate in fluids. Xiaolei Peng and coworkers from the University of Texas, US and Tsinghua University in Beijing, China, fabricated polystyrene microparticles that were half-coated with gold and suspended in a solution of water and 0.2 mM of cetyltrimenthylammonium chloride (CTAC). When illuminated with a defocused laser beam, asymmetric optical absorption and heating leads to a temperature gradient and an opto-thermoelectric field that propels the microparticles in the direction of the temperature gradient. Swimming velocities of up to nearly 20 µm/s were measured for 2.1 µm-sized particles illuminated with a 1064nm-wavelength laser beam with a low optical intensity of 0.03 mW/μm^2^. The use of a second focused laser beam makes it possible to rotate the microparticles and thus control the direction of motion.

## Introduction

Microswimmers represent a class of micromachines that are able to convert external chemical, acoustic, or electromagnetic energy to swimming motion^1^. Since the first demonstration of catalytic motors in 2004^2^, microswimmers have opened up avenues for diverse biomedical applications, including targeted drug delivery^3,4^, precision nanosurgery^5,6^, and diagnostic sensing^7,8^. In the past decade, self-propelled microswimmers powered by local chemical energy (e.g., the catalytic decomposition of hydrogen peroxide, which generates hydrogen bubbles for self-propulsion) have been widely studied^9–12^. Recently, fuel-free microswimmers driven by light fields^13–21^, magnetic fields^22–24^, electric fields^25,26^, and ultrasonic fields^27–29^ have been developed to expand the applications^30,31^. In particular, light-driven microswimmers show distinct advantages, as light allows the remote control of swimmers with high spatial and temporal resolution^14^. Though many optical manipulation techniques, such as optical tweezers, have been employed to transport micro-particles^32–34^, light-driven microswimmers represent exciting advancements with diverse transport modes (e.g., schooling, gravitaxis, and phototaxis)^35–37^, adaptive responses to ambient environments and easy light-swimmer coupling, enabling applications in biomedicine, sensing and environmental remediation^38–40^.

The light-driven self-propulsion of Janus particles paved the way towards optical microswimmers. It has been demonstrated that Janus particles can swim along their self-generated temperature or chemical gradient because of thermophoresis or diffusiophoresis^41–43^. However, the swimming direction of a particle becomes random at a longer time scale due to its rotational Brownian motion. To enable the long-term directional control of swimmers, photon nudging was proposed as one of the navigation strategies in which one would monitor the rotational Brownian motion of a swimmer and launch it once the swimmer was aligned with the target direction^15^. To improve the efficiency, active navigation strategies have been proposed to steer microswimmers. For example, hydrodynamic interactions between catalytic Janus particles and solid boundaries or topological features are exploited to quench Brownian rotation^10,44,45^. Another approach is to apply magnetic fields to magnetic swimmers to overcome the thermal rotation and guide the swimming direction^46–48^. Mimicking the phototaxis of motile photosynthetic microorganisms, artificial phototactic microswimmers are capable of sensing the light gradients in chemical fuels or critical mixtures for directional transport^38,49^. However, the global field for orientation control in the existing microswimmer system limits the development of a parallel platform for multitasking.

Inspired by the “run-and-tumble” behaviours of *E. coli* cells^50^, we demonstrate a category of all-optical microswimmers based on Janus particles in an optothermally generated electrical field^51–55^—opto-thermoelectric microswimmers—that exhibit coordinated actuation and navigation driven by two laser beams. Specifically, asymmetric light absorption of a Janus particle irradiated by an expanded laser beam leads to a self-generated temperature gradient and the resultant opto-thermoelectric field, which propels the particle directionally along the temperature gradient. With a second focused laser beam, we further propose triggering the in-plane rotation of individual Janus particles using a localised and dynamic opto-thermoelectric field under asymmetric optical heating. A stable particle rotation is achieved because of the balance among the thermoelectric force, optical force, and Stokes drag force. The optomechanical effects under temperature-gradient-induced electrical fields enable active optical navigation of the Janus particles without a complex design of the substrates or light profiles. By optically switching the propelling and rotating states of the Janus particles using a feedback control algorithm, we achieve the all-optical actuation, navigation, and target delivery of opto-thermoelectric microswimmers in fuel-free fluidic environments. We elucidate the working mechanisms by coupling experiments with theoretical analyses and simulations.

## Results

### Concept and design

**Fig. 1:**
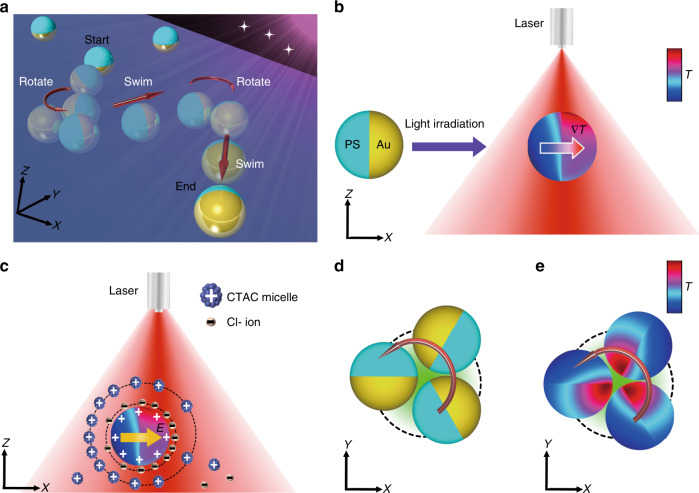
Conceptual design for optical driving and steering of opto-thermoelectric microswimmers. **a** Under light fields, PS/Au Janus particles are set to swim and rotate alternatively to follow a predefined path. **b** Upon light irradiation on a Janus particle, a temperature gradient ∇*T* pointing from the PS side to the Au side is generated on the particle surface due to the asymmetric absorption of PS and Au. **c** Once the Janus particle is dispersed in a 0.2 mM CTAC solution, a thermoelectric field is induced to drive the Janus particle along the temperature gradient. The white “+” symbols indicate the positively charged surface. In **b**, **c**, the asymmetric heating and thermoelectric field under a defocused laser beam are shown in the X–Z plane. **d** Schematic illustration and **e** asymmetric heating of the Janus particle when set to rotate (as shown by the maroon arrow) in the X–Y plane by another focused laser beam (indicated by the green region surrounded by a dashed circle). In **d**, **e**, the defocused laser beam is switched off

The design concept of the opto-thermoelectric microswimmers is illustrated in Fig. 1. The microswimmers are driven to swim and rotate alternatively through opto-thermal-electrical coupling under light fields, which allows well-defined guidance and transportation to a target (Fig. 1a). To enable photon-to-phonon energy conversion, we fabricated opto-thermoelectric swimmers by half-coating a thin Au layer on the surface of polystyrene (PS) beads (see Materials and Sample Preparation in Methods for details). Upon light irradiation, the absorbance difference between PS and Au creates a geometry-directed temperature gradient ∇*T* on the PS/Au Janus particle surface (pointing from the PS side to the Au side, as illustrated in Fig. 1b). To convert the thermal energy into mechanical energy, we dispersed the Janus particles in a water solution with 0.2 mM cetyltrimethylammonium chloride (CTAC, critical micelle concentration: 0.13 mM), where the Janus particles became positively charged due to the adsorption of CTAC surfactant and a thermoelectric field *E* was generated due to the different Soret coefficients between the CTAC micelles and counter Cl- ions (see Fig. 1c). Driven by the thermoelectric field, the Janus particles migrate along the PS-to-Au direction once they are irradiated by a defocused laser beam, which is termed the swimming state. However, it should be noted that thermal fluctuations will change the orientation of the Janus particles, causing the particles to drift off their courses during migration. To maintain the course to the target, we switched off the defocused laser beam and used another focused laser beam to trap and rotate the Janus particles for re-orientation (termed the rotation state, see Fig. 1d, e). Once the Janus particles reached the desired orientation, we turned off the focused laser beam and turned on the defocused laser beam to bring the Janus particles back to the swimming state. The two-state switching provides the possibility to design active navigating microswimmers for a variety of functionalities.

### Opto-thermoelectric swimming

**Fig. 2:**
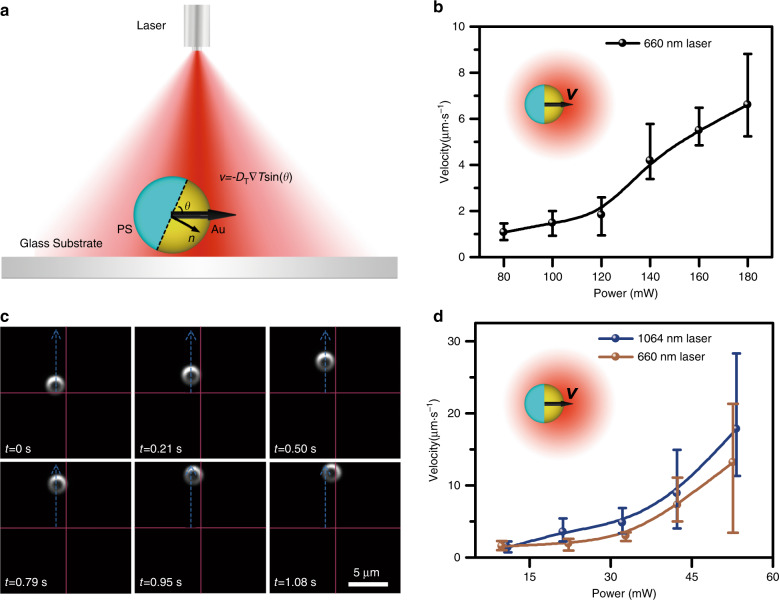
Opto-thermoelectric swimming of PS/Au Janus particles under a defocused laser beam. **a** Schematic illustration of the swimming mechanism. The velocity is directed from the PS hemisphere to the Au-coated hemisphere. **b** Swimming velocity as a function of the optical power for 5 µm PS/Au Janus particles. A 660 nm laser beam with a beam size of 31 µm was applied to drive the swimming. **c** Time-resolved images of a swimming 2.1 µm PS/Au particle. A 1064 nm laser beam with a beam size of 31 µm and a power of 32 mW was applied to drive the swimming. **d** Swimming velocity as a function of the optical power for 2.1 µm PS/Au Janus particles. Two different laser beams, i.e., a 1064 nm laser beam with a beam size of 45 µm and a 660 nm laser beam with a beam size of 45 µm, were applied to drive the swimming. The insets of **b**, **d** show a PS/Au Janus particle driven to swim under a defocused laser beam. All the aforementioned beam sizes were obtained by experimental measurement

Directed motion of opto-thermoelectric microswimmers is achieved when they are illuminated by a defocused laser beam, which provides an “energy pool” for the Janus particles. As shown in Fig. 2a, a PS/Au Janus particle is driven along the self-generated temperature gradient, which is termed self-thermophoresis. A general equation to evaluate the velocity of directed motion caused by self-thermophoresis, or the thermophoretic swimming velocity, is written as ***v*** = −*D*_T_∇*T*sin(*θ*), where *D*_T_ is the thermodiffusion coefficient, ∇*T* is the temperature gradient, and *θ* is the angle between the PS/Au interface and the substrate. In the CTAC solution, self-thermophoresis arises from the thermoelectric effects (see Fig. 1c and Supplementary Note S1), leading to a negative *D*_T_ and driving the Janus particle towards the temperature gradient, i.e., from the PS hemisphere to the Au-coated hemisphere.

Figure 2b shows the swimming velocity of 5 µm PS/Au Janus particles as a function of the optical power under the illumination of a defocused 660 nm laser beam. For a spherical Janus particle, the swimming velocity can be approximated as $\it v = - D_{\mathrm{T}}\frac{{{\Delta}T}}{{3R}}{\mathrm{sin}}\left( \theta \right)$^41^, where Δ*T* is the temperature increase and R is the radius of the particle. Interestingly, although Δ*T* is proportional to the laser power, the swimming velocity is nonlinearly proportional to the laser power, which is due to a larger *θ* for the Janus particles swimming at higher optical power (see Supplementary Fig. S1). The larger *θ* is due to the better alignment under a stronger thermoelectric field, the physical mechanism of which is detailed in the following section “Orientation control”. We further demonstrated that the directed motion of a single 2.1 µm PS/Au Janus particle could be induced by a defocused laser beam of the optothermal responsive wavelength at different laser powers, as shown in Fig. 2c, d and Supplementary Movie S1. The chamber thickness can be further reduced to stabilise the fluidic flow and to facilitate the directional transport of Janus particles with smaller sizes down to 0.5 μm (the smallest size of the Janus particles we tested). In addition, we used two different wavelengths, 660 nm and 1064 nm, to verify that the swimming mechanism works for different laser wavelengths that can optically heat the Janus particles. The swimming velocity upon the irradiation of the 1064 nm laser beam was higher than that upon the irradiation of the 660 nm laser beam because the particle had stronger light absorption at 1064 nm (see Supplementary Fig. S2). A similar nonlinear relation between the swimming velocity and the optical power is observed, and a swimming velocity of 10 µm·s^−1^ can be achieved with an optical power 4–5 times lower than that of single 5 µm PS/Au Janus particles. The lower power requirement is mainly ascribed to a higher surface temperature gradient for the Janus particles with smaller sizes (∆*T* ∝ *R*^−1^ and ∇*T* ∝ *R*^−2^)^41^.

### Orientation control

**Fig. 3:**
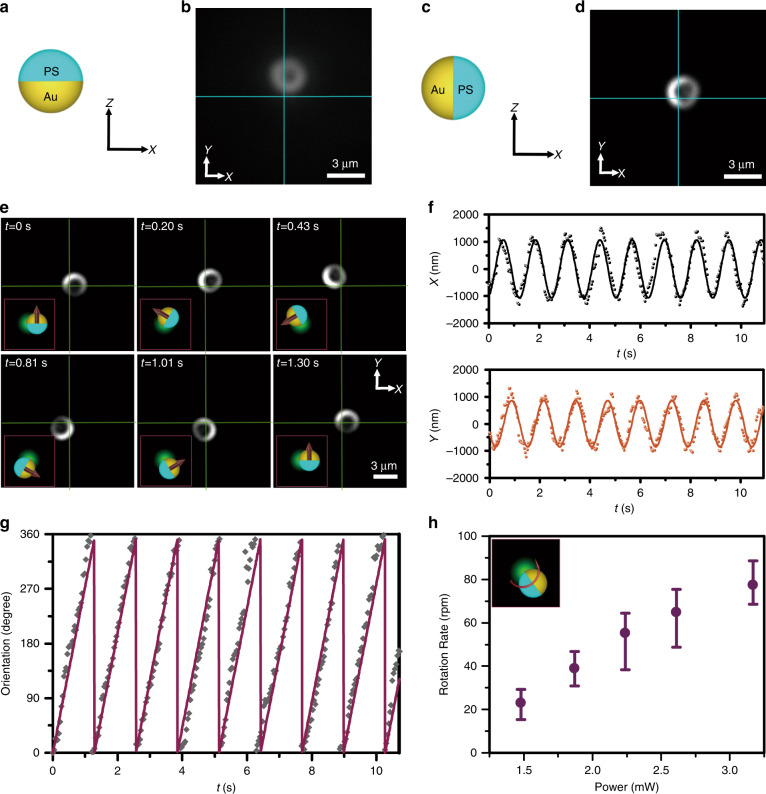
Orientation control of PS/Au Janus particles with a focused laser beam. **a** Configuration and **b** corresponding dark-field image of a free 2.7 µm PS/Au Janus particle in the X–Z plane. **c** Configuration and **d** corresponding dark-field image of a rotating 2.7 µm PS/Au Janus particle in the X–Z plane. **e** Time-resolved dark-field images of the rotation of a 2.7 µm PS/Au Janus particle. The half-cyan, half-golden particles in the insets illustrate the corresponding configurations, while the maroon arrows in the insets illustrate the orientations. The green spot in the insets represents the laser beam (with a wavelength of 532 nm). **f** Displacement of the centre of the 2.7 µm Janus particle as a function of time. The centre of the beam is set as the origin of the coordinates. The fitting sinusoidal curves indicate a circular rotation. **g** Orientation evolution of the 2.7 µm Janus particle as a function of time. The fitting sawtooth wave indicates a consistent steering of the orientation. **h** Rotational rate as a function of the optical power for 2.7 µm PS/Au Janus particles. In **a**–**d**, for a free Janus particle, no boundary at the particle hemisphere was observed in the dark-field optical image because the Au-coated part tended to align with the direction of gravity. In contrast, when in-plane rotation of the Janus particle was initiated, the PS-Au interface became perpendicular to the substrate due to the coordinated effect of the thermoelectric force and the optical force. An asymmetric ring was observed in the dark-field optical image, with the brighter half-ring corresponding to the Au coating owing to its stronger optical scattering. The inset illustrates the rotation under a green laser beam (with a wavelength of 532 nm). The laser beam size on the sample plane is 2.65 µm for **e**, **h**. A power of 1.9 mW was applied for rotation in **e**

Ideally, opto-thermoelectric swimmers can be driven directionally along the self-generated temperature gradient. However, the orientation of the Janus particles is randomly changed by thermal fluctuations. As a result, the Janus particles drift off their courses during directional migration. Different from photon nudging, which utilises thermal fluctuations as a passive mechanism for particle orientation control^15^, we use a second focused laser beam to achieve active rotation of a Janus particle and thus its swimming direction in a more efficient way. It should be noted that the focused laser beam used for particle rotation cannot operate simultaneously with the defocused laser beam that drives the swimming, as co-irradiation will modify the temperature distribution on the particle surface and disrupt the swimming behaviour. It is noted that the PS/Au interface can be clearly observed during the rotation because the interface is almost aligned perpendicular to the substrate. In contrast, the interface cannot be clearly observed for free Janus particles since the Au-coated part tends to approach the substrate due to the effect of gravity (see Fig. 3a–d). Figure 3e presents time-resolved dark-field optical images of the anti-clockwise rotation of a single PS/Au Janus particle irradiated by a focused 532 nm laser beam. The orientation of the particle can be determined through the difference in scattering intensity between Au-coated (bright part) and PS (dark part) hemispheres. From the dark-field images, we can see that there is an offset between the centre of the particle and the centre of the beam. It should also be noted that the rotational direction can be either clockwise or anti-clockwise, depending on the orientation of the Janus particle when the particle approaches the laser beam. Once a Janus particle is set to rotate around the laser beam, the rotational direction can be stably maintained.

For quantitative analysis, we tracked the rotating Janus particle and extracted the real-time position and orientation data (see Methods for the particle tracking algorithms). Figure 3f plots the displacement between the particle centre and the beam centre along the X and Y axes, respectively. The particle rotates in circles around the centre of the beam without any hopping behaviour across the centre of the beam, as indicated by the sinusoidal curve fit. We define the orientation of the Janus particle as normal to the PS/Au interface (indicated by the red arrows in the insets of Fig. 3e). The time evolution of the angle between the orientation of the Janus particle and the Y axis is given in Fig. 3g, which follows a sawtooth line shape and reveals a constant rotation speed of 40 rpm at an optical power of 1.9 mW. The rotation speed can be tuned by controlling the laser power.

As shown in Fig. 3h, the rotation speed increases from 40 to 80 rpm when the laser power increases from 1.9 mW to 3.2 mW. However, when the laser power is further increased, a strong heating effect occurs, causing thermal damage to the Janus particles without any bubble formation. It is also noted that the rotational speed is size-dependent. As shown in Fig. S3 and Supplementary Movie S2, the rotation speed of the 5 µm PS/Au Janus particles is much slower than that of the 2.7 µm PS/Au Janus particles due to the larger size and stronger friction. It should be noted that a larger beam size is used for the rotation of the 5 µm PS/Au Janus particles to prevent overheating of the particles.

**Fig. 4:**
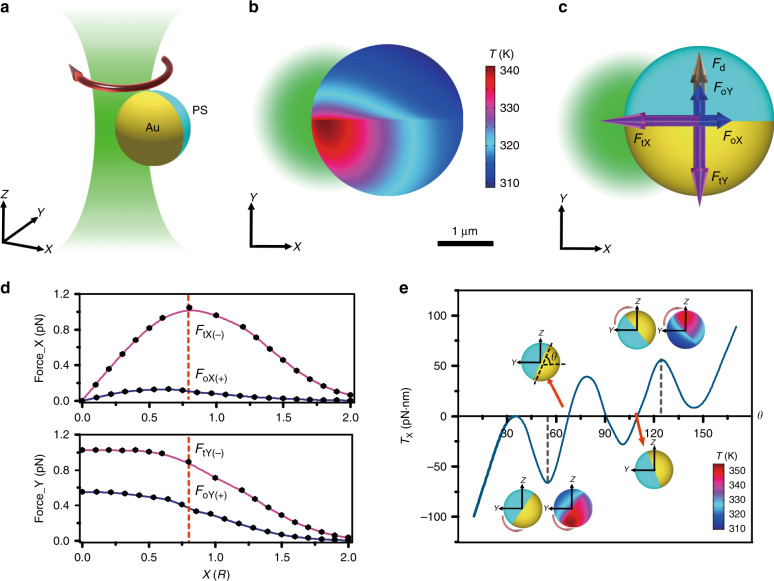
Mechanism for orientation control of PS/Au Janus particles. **a** Schematic illustration of a PS/Au Janus particle rotating under a focused green laser beam. **b** Simulated temperature profile and **c** force analysis of a 2.7 µm PS/Au rotating under a focused green laser beam. The orientation is parallel to the Y axis, and the offset between the centre of the particle and the centre of the beam is equal to 0.8 *R*. The laser beam propagates along the negative Z direction. The power of the green laser beam is set as 2 mW. **d** Calculated X and Y components of the thermoelectric force and optical force as a function of the offset (the origin is at the centre of the beam). The red dashed lines indicate the balance position *X* = 0.8 *R*. **e** Calculated thermoelectric torques in the balance position (*X* = 0.8 *R*) as a function of *θ*, which is the angle of the PS/Au interface relative to the substrate (X–Y plane). The insets of **e** depict the corresponding configurations and temperature profiles at certain *θ* (indicated by dashed lines or arrows)

To understand the role of the thermoelectric force during the in-plane rotation, we simulated the temperature distribution on the surfaces of the PS/Au Janus particles (see Methods for the simulation setup). We considered a 2.7 µm PS/Au particle rotating under a focused 532 nm laser beam. For simplicity of denotation, the origin of the coordinates was defined at the centre of the beam, with the X axis parallel to the PS/Au interface and Z axis parallel to the beam propagating direction, as shown in Fig. 4a. To reproduce the experimental conditions in the simulation setup, a green laser beam with a diameter of 2.65 µm and an optical power of 2 mW was irradiated on a 2.7 µm PS/Au particle (see Supplementary Fig. S4), while an offset of 0.8 *R* was set between the centre of the particle and the centre of the beam (*R* is the particle radius; see Supplementary Fig. S5). Compared with the ambient temperature, a maximum temperature increase of ~50 K was observed for the 2.7 µm Janus particle, with the hot spot located at the Au-coated hemisphere and close to the centre of the beam (Fig. 4b). Since the thermoelectric force in the CTAC solution points from the cold region to the hot region (see Supplementary Note S1 for details), the X component of the thermoelectric force *F*_tX_ will attract the particle to the centre of the beam, while the Y component of the thermoelectric force *F*_tY_ will drive the particle towards the Au-coated hemisphere, as shown in Fig. 4c. However, because of the existence of the Au coating and resultant strong light scattering, the X component of the optical force *F*_oX_ will repel the particle outward from the laser beam, while the Y component of the optical force *F*_oY_ will drive the particle towards the PS hemisphere, as shown in Fig. 4c. In addition to asymmetric heating, the thermoelectric force must be directed from cold to hot regions to achieve in-plane rotation. Otherwise, the optical force cannot be balanced, and the particle will be repelled away. To verify this hypothesis, we changed the CTAC solution to phosphate buffer saline (PBS) solution, in which the thermoelectric force directs from the hot to the cold region. We can observe that the Janus particles were either repelled away (Supplementary Movie S3) or wobbling (Supplementary Movie S4) under the laser beam. It should be noted that microengines driven by optical forces^56,57^ and self-diffusiophoresis^19^ can achieve stable rotation as well. However, the requirement for precise temperature control the mixture criticality design, and the challenge in achieving a tuneable rotation rate impede further development into two-state swimmers, as reported here.

For a comprehensive understanding of the rotation dynamics, we calculated both the thermoelectric force and the optical force (see Supplementary Note S1 and Methods for details)^52^. Fundamentally, the asymmetric-heating-induced thermoelectric force drives the rotation, *i.e*., it provides the centripetal force along the X axis and overcomes the optical force and the Stokes drag force along the Y axis for stable rotation. Figure 4d shows the X and Y components of both the thermoelectric force and the optical force as a function of the offset (the origin is at the centre of the beam). At the balance position (*X* = 0.8 *R*), the centripetal force is provided by the synergistic effect of *F*_tX_ (1.05 pN), *F*_oX_ (0.11 pN), and a static friction force *F*_r_ (0.84 pN) (see Supplementary Note S2 for detailed calculations). Simultaneously, *F*_tY_ (0.89 pN) is balanced by the sum of *F*_oY_ (0.39 pN) and the effective Stokes drag force *F*_d_ (0.61 pN, calculated based on a friction torque analysis; see Supplementary Note S2), explaining the constant rotational speed observed in the experiments. It should be noted that the thermoelectric force cannot overcome the strong optical force along the Z axis, and a substrate is required to achieve the force balance (Supplementary Fig. S6).

We emphasise that the PS/Au interface is almost perpendicular to the substrate during the in-plane rotation of the Janus particles. To understand this self-alignment behaviour, we defined the angle between the PS/Au interface and the substrate as *θ* and calculated the out-of-plane thermoelectric torque *T*_tX_ with the particle centre as the reference point. Figure 4e plots the calculated thermoelectric torque *T*_tX_ as a function of *θ* at the balance position (*X* = 0.8 *R*). The calculated thermoelectric torque (10–100 pN·nm) is comparable to the optical torque for optically propelled micro-rotors^56^, while the required optical intensity in our experiments is 100 times lower. There are two energetically favourable equilibrium orientations, *i.e*., $\theta = \frac{3}{8}\pi \,{\mathrm{or}}\,\frac{5}{8}\pi$, where the thermoelectric torque is zero and restoring thermoelectric torques are induced once the orientation changes. This self-alignment behaviour arises from the asymmetric heating of the Janus particle along the Z axis. As shown in the insets of Fig. 4e, at small *θ*, the hot region (Au-coated hemisphere) is located at the bottom, leading to a negative thermoelectric torque that rotates the particle anti-clockwise. At large *θ*, the hot region (Au-coated hemisphere) switches to the top, leading to a positive thermoelectric torque that rotates the particle clockwise. This observation explains why the PS/Au interface is almost perpendicular to the substrate and can be clearly observed during the rotation process. It should be noted that the optical torque will also contribute to the self-alignment phenomenon. The optical torque is comparable to that of the thermoelectric torque near 90°, while it is significantly smaller near 0° or 180° (see Supplementary Fig. S7).

### Feedback control method

**Fig. 5:**
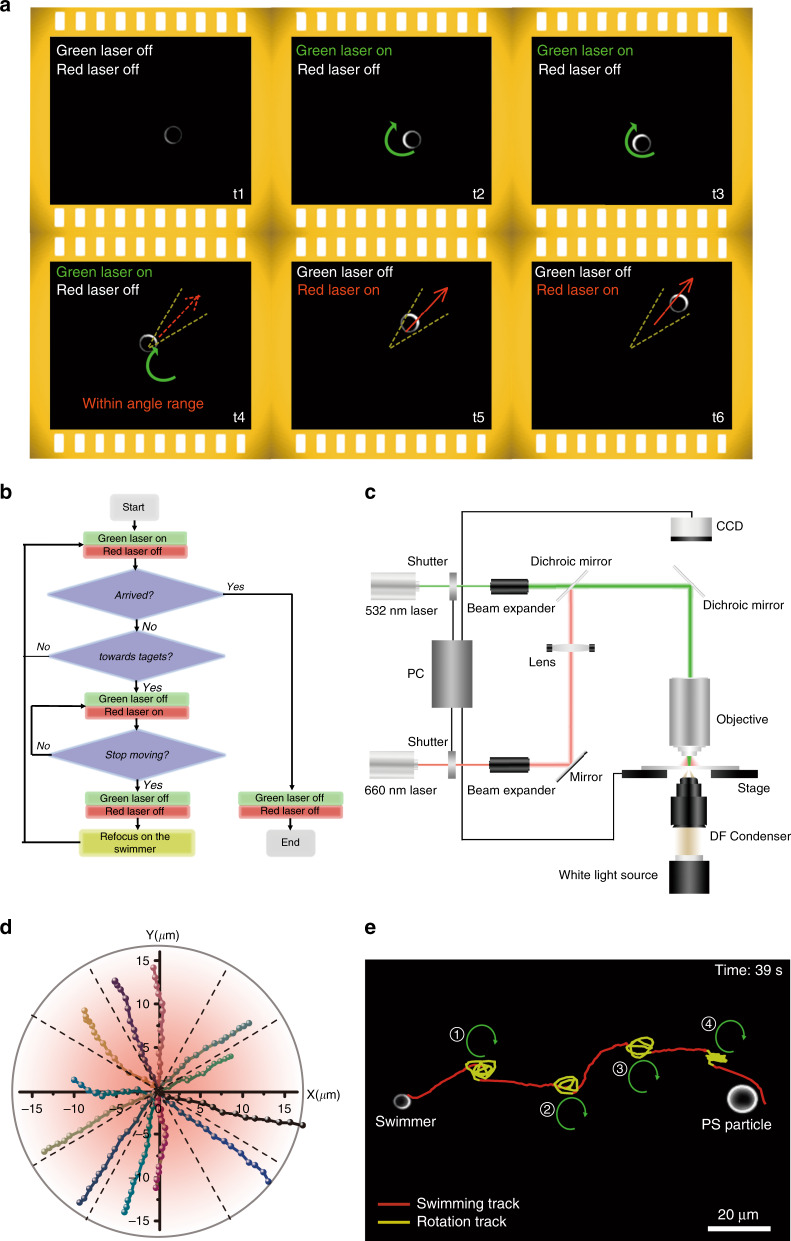
Directional swimming and targeted transportation of PS/Au Janus particles with a feedback control method. **a** Schematic illustration of directional swimming with feedback control on the experimentally recorded images, where a focused green laser beam and a defocused red laser beam were employed for navigating and driving the swimming, respectively. **b** Flow chart of the feedback control method. **c** Optical setup and mechanical layout for the feedback control method. **d** Trajectories of 5 µm PS/Au Janus particles swimming in different directions. **e** Targeted delivery of a 5 µm PS/Au Janus particle to a 10 µm PS particle. A 5 µm 532 nm laser beam with a power of 2.6 mW was used to drive the rotation, while a 660 nm laser beam with a beam size of 31 µm and a power of 160–200 mW was applied to drive the swimming

The ability to rotate a Janus particle for orientation control provides the possibility to steer the swimming direction. Specifically, we establish a feedback algorithm to enable the active navigation functionality. The key idea is to optically switch the microswimmer between the rotation state and the swimming state through identification of the offset between its course and target until it reaches its target, as shown in Fig. 5a. To accomplish closed-loop control, a computer programme is developed to track the real-time position and orientation of a given Janus particle and coordinate the control system automatically (see Fig. 5b, Methods, and Supplementary Note S3 for more details). The experimental setup is illustrated in Fig. 5c, in which two computer-controlled shutters are used to control the on/off states of two individual laser beams. As the first demonstration, we succeeded in driving the directional swimming of Janus particles within twelve pre-designed angle ranges (at an interval of 30°), as shown in Fig. 5d and Supplementary Movie S5. It should be noted that an increase in rotation speed will reduce the control accuracy of the swimming direction, considering the long response time of the shutters (~500 ms) and the low frame rate of the CCD camera (12 frames per second or fps) in our current setup. In other words, the accuracy of the feedback control can be significantly improved using a higher-frame-rate CCD camera and shutters of shorter response time (e.g., acousto-optic modulators).

To demonstrate active navigation, we applied the feedback control algorithm for the targeted transportation of a 5 μm PS/Au Janus particle, as shown in Fig. 5e and Supplementary Fig. S8. A 10 μm PS particle was fixed on the substrate as the target, which was 110 µm away from the swimmer. At the beginning, the opto-thermoelectric swimmer was set to the swimming state, with its orientation being tracked in real time. When the deviation between the swimming direction and the designed path exceeded ±15°, the stage would move automatically to realign the microswimmer to the position of the green laser beam, inducing the rotation state until the deviation was smaller than ±15°. Then, the swimmer was set back to the swimming state to approach the target. Compared with microswimmers driven by global fields^22,37,50^, our focused laser beam for particle rotation can control individual swimmers for independent operation, which has advantages in multi-tasking application scenarios where different swimmers are required to undergo different paths for various targets. Through the two-state switching, the opto-thermoelectric swimmer directionally migrates over 110 µm to reach the target in 39 s after four re-orientations. The navigation efficiency can be doubled (i.e., ~18 s to migrate over 110 µm) if we use a fast camera (with 100 fps) and acousto-optic modulators as laser shutters (which feature a response time <100 ns). We can see that not all the swimming directions can perfectly fall in the predesigned angle range during delivery due to the thermal fluctuations. However, our algorithm can amend those errors through multiple-loop control (see Supplementary Fig. S9). It is worth noting that optical tweezers can also deliver micro-objects to a target with a single focused laser beam in a “simpler” manner. Nevertheless, the high operational power of optical tweezers can cause photon damage to objects and has limitations in applications such as drug delivery and target drug injection. Herein, our technique can precisely deliver the metal-coated Janus particle to the target *via* two working modes at a low operation power. Thus, opto-thermoelectric microswimmers are promising for carrying drug molecules on non-metallic parts and precisely delivering them to target cells^58^.

## Discussion

In summary, we have developed opto-thermoelectric microswimmers with all-optical actuation and navigation by harnessing opto-thermal-electrical coupling at the Janus particles. The heat generated from the light-irradiated Janus particles creates a thermoelectric field, which propels the particles directionally without chemical fuel. The orientation of the microswimmers is steered by asymmetric-heating-induced in-plane rotation of the Janus particles under a focused laser beam. By alternatively switching the Janus particles between propulsion and rotation states with a feedback control algorithm, we have achieved new functionalities of the microswimmers, including precise positioning and automatic navigation. This active navigating and opto-thermoelectric control system can be further exploited to develop intelligent microrobots with functionalities such as target location, path selection, and speed optimisation. Moreover, precise orientation control using a local laser beam provides the possibility to develop a parallel microswimmer system for multitasking.

## Materials and methods

### Materials and sample preparation

PS spheres were purchased from Bangs Laboratories. Monolayers of dielectric particles, including 2.1 µm, 2.7 µm, and 5 µm PS microspheres, were created on glass substrates through spin coating. Then, a layer of Au films with a thickness of 25–30 nm was deposited on the monolayers of dielectric particles to fabricate the Janus microswimmers. The Janus microswimmers were detached from the glass substrates with a blade and dispersed in ethanol after sonication. The Janus swimmers were re-dispersed in a water solution of 0.2 mM CTAC for the experiments. In our experiments, the Janus particle suspensions were confined in a chamber with a thickness of 120 μm.

### Optical setup

A 532 nm diode-pumped solid-state laser (Laser Quantum) was expanded with a home-built 3× beam expander, directed into a commercial upright microscope (Nikon), and focused by a 20X (Nikon, NA 0.5) or a 100X (Nikon, NA 0.6) objective, creating a focused laser beam ~5 µm above the glass substrate. A 660 nm diode-pumped solid-state laser (Laser Quantum) or a 1064 nm diode-pumped solid-state laser (CrystaLaser) was expanded with another home-built 3× beam expander and directed into the commercial upright microscope. For the 660 nm laser or the 1064 nm laser, a 30 cm lens was inserted between the beam expander and the upright microscope to create defocused laser beams with desired sizes on the glass substrate. All the laser power values were directly measured above the samples. For the 2.1 µm and 2.7 µm PS/Au Janus particles, the 100X objective was used, while for the 5 µm PS/Au Janus particles, the 20X objective was used. A fast CCD camera (Andor, 100 fps) was integrated into the upright microscope to record the rotation and swimming of Janus microswimmers under dark-field illumination.

### Particle tracking

The recorded movies were converted into a series of greyscale images, with pixel intensities ranging from 0 to 255. The greyscale images were then converted to binary images by setting an intensity range. The intensity range was optimised for each set of movies so that the Janus particles could be clearly resolved in the binary images. The positions of the particles were obtained by fitting the central dark area of the Janus particles with a circle and extracting the coordinates of the central point. The orientations of the Janus particles were obtained based on the positions of the particles and the mean position of the bright part of the particles.

### Feedback control

The feedback control method was based on a homebuilt LabVIEW programme (LabVIEW 18.0, National Instruments). The dark-field images were captured by a CCD camera (Lumenera, 12 fps) with 1392×1040 pixels. The captured images were transformed into binary images by a threshold operation. The Janus particles as well as the target particles were first processed by the IMAQ fill hole module to transform the hollow particles into solid ones. The IMAQ particle filter module was then applied to distinguish two kinds of particles by setting different thresholds for the sectional areas. After filtering the particles, the geometrical centres of the Janus particles and the target particles, ***P***_sc_(*t*)/***P***_t_(*t*), could be directly obtained by the IMAQ particle analysis module. For the brightest point of the Janus particles ***P***_sb_(*t*), a region of interest around a given Janus particle was cropped to avoid incorrectly obtaining the brightest point of other particles, and ***P***_sb_(*t*) was obtained by using the IMAQ Image-To-Array module. All these coordinates were updated at the beginning of each loop, and the two angles (*θ*(*t*), *α*(*t*)) were calculated accordingly. Based on the algorithms mentioned in the feedback loop, the corresponding signals were directly sent to the stage and two shutters *via* universal serial bus connections to coordinate the stage movement and/or laser inputs automatically.

### FEM simulation

A finite-element solver (COMSOL Multiphysics 5.2) was used to simulate the temperature profile of a Janus particle. In the simulation, the Janus particle was placed above the glass substrate with a 30 nm gap. The incident light was a Gaussian beam propagating in the negative Z direction. The physics model involves electromagnetic waves and heat transfer in solids and fluids. The multi-physics coupling includes an electromagnetic heat source, boundary electromagnetic heat source, and temperature coupling. The boundary conditions for the electromagnetic wave and heat transfer were set as the scattering boundary and room temperature, respectively. The background medium was set as water (refractive index of 1.33).

### FDTD simulation

The finite-difference time-domain method (Lumerical Inc.) was applied for the simulation of optical forces and torques. The reflective index of the dielectric material for a Janus particle was set as 1.45, and the permittivity of the Au films was taken from Johnson and Christy^59^. The optical forces and torques were calculated through the Maxwell stress tensor (MST) at an excitation wavelength of 532 nm. The mesh size in the simulation was set as 5 nm.

## Supplementary information

Supplementary Information

Swimming of a 2.1 μm PS/Au Janus particle in 0.2 mM CTAC solution

Rotation of 2.7 μm and 5 μm PS/Au Janus particles

Rotation of a 2.7 μm PS/Au Janus particle in CTAC solution and repelling of a 2.7 μm PS/Au Janus particle in 2% PBS solution

Rotation of a 5 μm PS/Au Janus particle in 0.2 mM CTAC solution and wobbling of a 5 μm PS/Au Janus particle in 10% PBS solution

Swimming of 5 μm PS/Au Janus particles in different directions in 0.2 mM CTAC solution

## Data Availability

The data that support the plots within this paper and other findings of this study are available from the corresponding author upon reasonable request. Supplementary information accompanies the manuscript on the Light: Science & Applications website (http://www.nature.com/lsa).
