# Supplementary material for: Opto-thermoelectric microswimmers
Source: Light Sci Appl. 2020 Aug 17;9:141. doi: 10.1038/s41377-020-00378-5 (PMC7429954; doi:10.1038/s41377-020-00378-5)
Supplement: Supplementary file 1 — Supplementary Information [file 41377_2020_378_MOESM1_ESM.docx]

Supporting Information

Opto-Thermoelectric Microswimmers

Xiaolei Peng,^1^^†^ Zhihan Chen,^1†^ Pavana Siddhartha Kollipara,^2^ Yaoran Liu,^1^ Jie Fang,^1^ Linhan Lin*,^3^ and Yuebing Zheng*^1,2^

^1^Materials Science & Engineering Program and Texas Materials Institute, The University of Texas at Austin, Austin, TX 78712, USA.

^2^Walker Department of Mechanical Engineering, The University of Texas at Austin, Austin, TX 78712, USA.

^3^State Key Laboratory of Precision Measurement Technology and Instruments, Department of Precision Instrument, Tsinghua University, Beijing 100084, People’s Republic of China

^†^These authors contributed equally to this work.

^*^Email: linlh2019@mail.tsinghua.edu.cn; ^*^E-mail: zheng@austin.utexas.edu

**Supplementary Movies**

Movie S1: Swimming of a 2.1 μm PS/Au Janus particle in 0.2 mM CTAC solution. (MOV)

Movie S2: Rotation of 2.7 μm and 5 μm PS/Au Janus particles. (MOV)

Movie S3: Rotation of a 2.7 μm PS/Au Janus particle in CTAC solution and repelling of a 2.7 μm PS/Au Janus particle in 2% PBS solution. (MOV)

Movie S4: Rotation of a 5 μm PS/Au Janus particle in 0.2 mM CTAC solution and wobbling of a 5 μm PS/Au Janus particle in 10% PBS solution. (MOV)

Movie S5: Swimming of 5 μm PS/Au Janus particles in different directions in 0.2 mM CTAC solution. (MOV)

**Supplementary Figures**

**Fig. S1: Swimming of 5 μm PS/Au Janus particles**


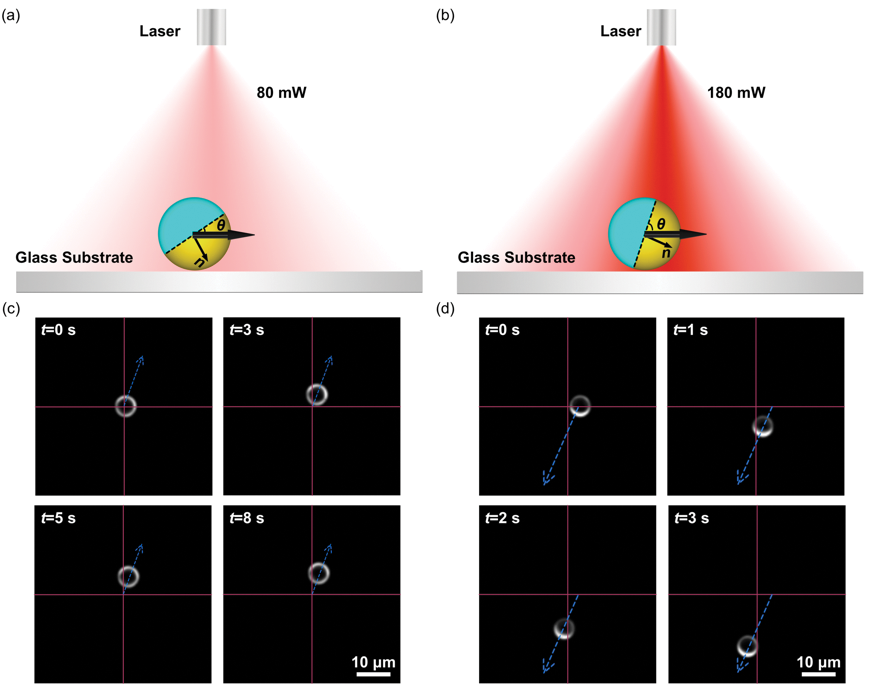


**Fig. S1: a** A small angle $\theta$ relative to the substrate in an optical power of 80 mW. **b** A large angle$\theta$ relative to the substrate under an optical power of 180 mW. **c** Time-resolved dark-field images of a Janus particle swimming in an optical power of 80 mW. **d** Time-resolved dark-field images of a Janus particle swimming under an optical power of 180 mW. In **a** and **b**, $\theta$is the orientation relative to the substrate. A lower contrast between the bright part and the dark part of the Janus particle in **c** implies a smaller angle $\theta$ in an optical power of 80 mW. At the higher power, the thermoelectric force will dominant over the scattering force, which tends to increase$\theta$. A 660 nm laser beam with a beam size of 31 μm was applied to drive the swimming.

**Fig. S2: Absorption cross-section of a 2 μm PS/Au Janus particle as a function of laser wavelength**

**

**

**Fig. S2:** The absorption cross section of a 2 μm Janus particle as a function of the wavelength of incident laser from 400 nm to1200 nm.

**Fig. S3: Rotation of 5 μm PS/Au Janus particles**


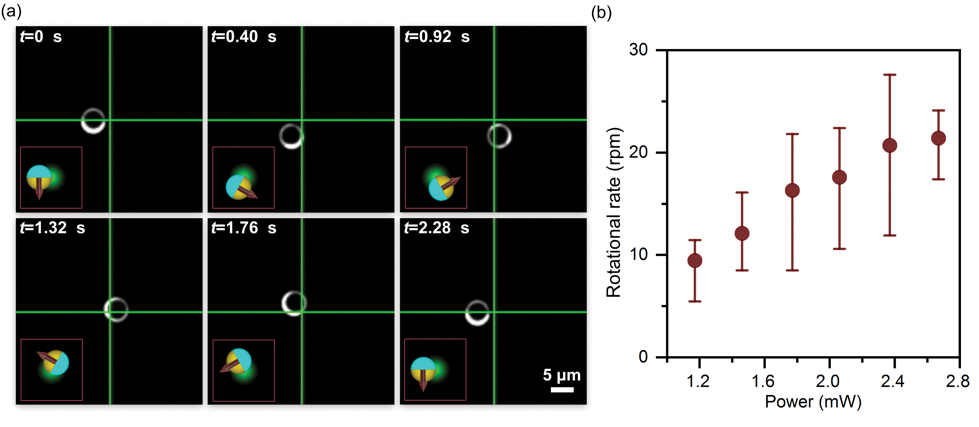


**Fig. S3:** **Rotation of 5 μm PS/Au Janus particles on a glass substrate under a focused laser beam.** **a** Time-resolved images of the rotation of a 5 μm PS/Au Janus particle. The particles of half cyan and half golden in the insets illustrate the corresponding configurations, while red arrows in the insets illustrate the rotational directions. The green spot in the insets represents the laser beam. **b** Rotational rate as a function of optical power for 5 μm PS/Au Janus particles. The green laser beam size is 5 μm on the sample plane for **a** and **b**. A power of 2.6 mW is applied for rotation in **a**.

**Fig. S4-S7: Mechanism for the rotation of PS/Au Janus particles**


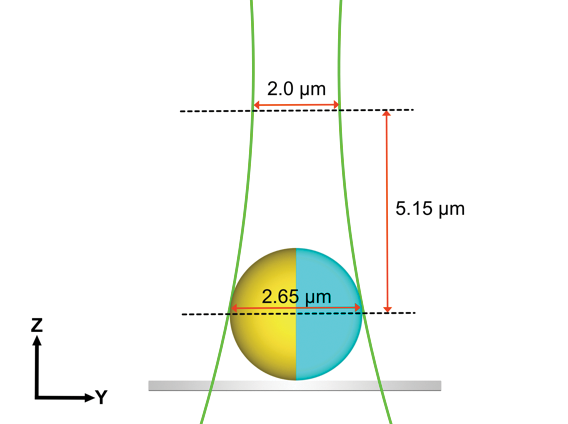


**Fig. S4:** Front view of the beam profile and geometric setup for a 2.7 μm PS/Au Janus particle rotating on a glass substrate. The beam waist is well above the particle. The orientation of the particle is parallel to the Y axis.


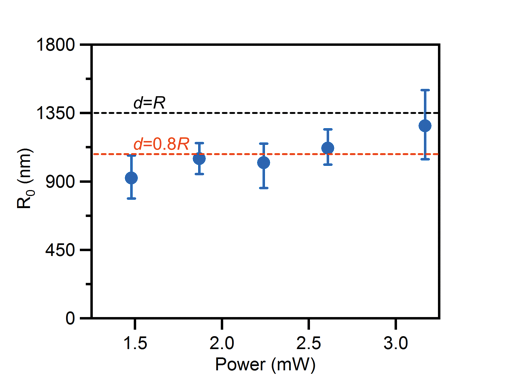


**Fig. S5:** The distance *R*_0_ between the center of the particle and the center of the beam as a function of optical power for 2.7 μm PS/Au Janus particles.


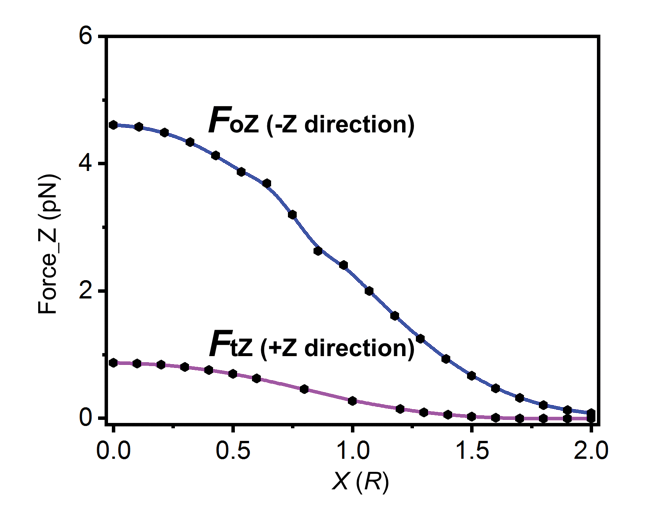


**Fig. S6:** Calculated thermoelectric forces and optical forces in the Z axis when scanning the particle along the X axis. The thermoelectric force along the plus Z direction is much weaker than the optical scattering force along the minus Z direction, so a substrate is required to confine the particle near the substrate in the Z direction.


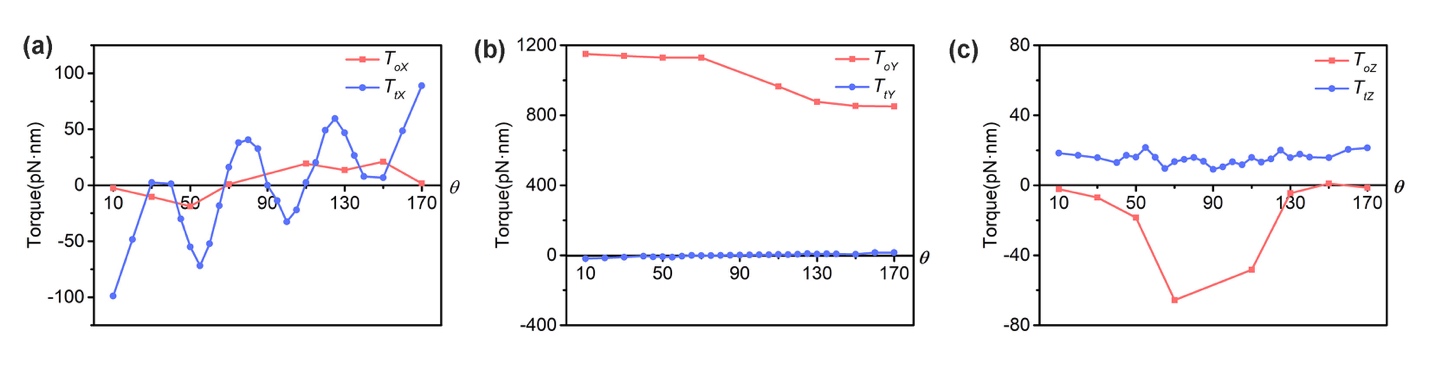


**Fig. S7:** Calculated optical and thermoelectric torques in the balance position (*X* = 0.8 R) as a function of the rotational angle $\theta$along **a** the X axis, **b** the Y axis, and **c,** the Z axis. Here, the reference point for torque calculation is the particle center. In **b**, a total of the optical torque and the thermoelectric torque is a positive torque of 1130 pN·nm (or 1.13 pN·μm), which is counterbalanced by a negative friction torque. The negative friction torque is caused by a static friction force of F_r_ (0.84 pN) along the +X axis at the bottom of the particle (*R* = 1.325 μm), as no rotation is observed experimentally with respect to the Y axis. The friction force is due to the substrate effect. In **c**, there is a negative total *T*_Z_ when the rotation angle is ~ 90 degree, which is the driving torque and leads to the continuous clockwise rotation along the Z axis.

**Fig. S8-S9: Targeted delivery of opto-thermoelectric microswimmers**

**
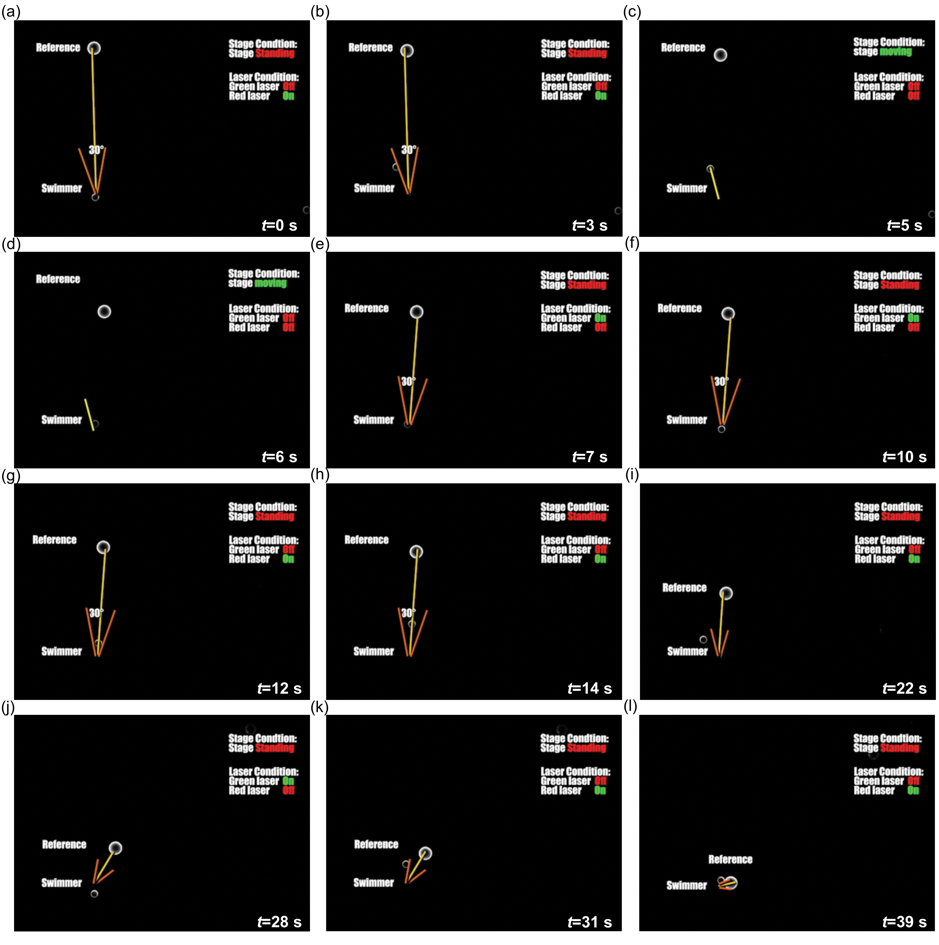
**

**Fig. S8:** Time-resolved images of long-range transport of a 5 μm PS/Au Janus particle to a 10 μm PS particle with a feedback control method.


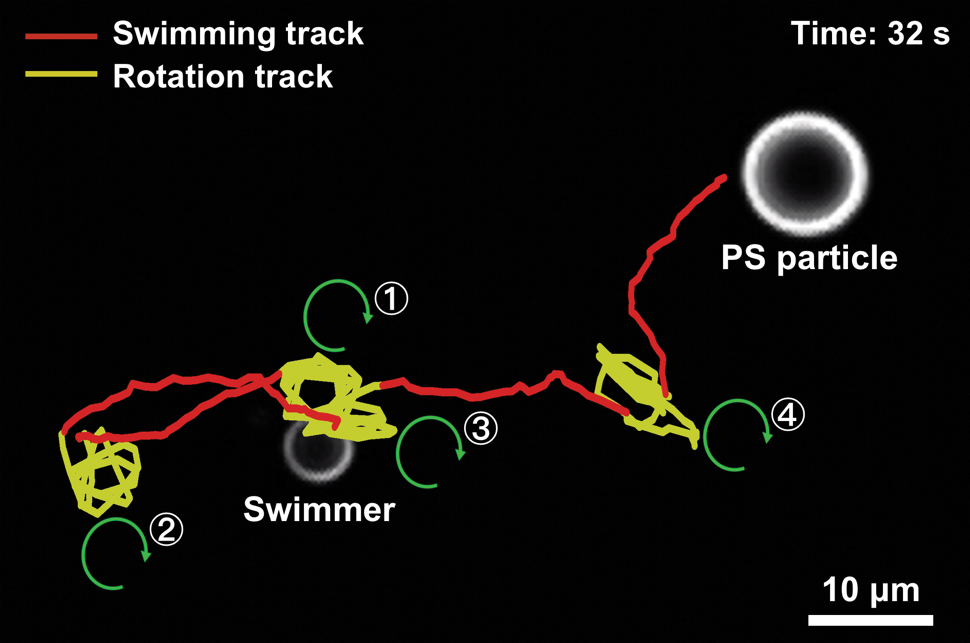


**Fig. S9:** Targeted delivery of a 5 μm PS/Au Janus particle to a 10 μm PS particle. In the first rotation track, the unstable rotation leads to a large deviation from the targeted path in the following swimming track, but the particle can still be transported to the target for the robust control method.

**Supplementary Notes**

**Note S1: Thermoelectric forces and torques on PS/Au Janus particles**

CTAC is introduced into the dispersion of Janus particles to generate opto-thermoelectric forces under light fields. CTAC will decompose into the cation CTA^+^ and the anion Cl^-^ ions in water. The cation (CTA^+^) has a positive hydrophilic end and a hydrophobic polymer chain, which enables it to form globules beyond a critical micellar concentration (cmc), known as micelles. The CTAC micelles will adsorb on the Janus particles and lead to positive charged particle surfaces. The CTAC micelles have a much larger Soret coefficient *S_T_* than that of Cl^-^ ions under a temperature field, resulting positive thermoelectric fields that direct the positively charged Janus particles toward the temperature gradient, *i.e.*, from the cold region to the hot region.

The thermoelectric force on a Janus particle is solely due to the tangential component of the thermo-electric field as the normal component of thermo-electric fields is neutralized by the screening effect of the Stern layer around the particle surface and the inherent charge on the particle surface. Assuming no effect from positive ions, the resultant *E* field is taken as

|  | $E_{T}=\frac{k_{B}T\nabla T}{e}\frac{\left[ S_{T_{2}}+\frac{Z_{1}S_{T_{1}}}{N_{\mathrm{agg}}} \right]}{1-\frac{Z_{1}S_{T_{1}}T}{N_{\mathrm{agg}}}}=S_{C}\nabla T$ | (1) |
| --- | --- | --- |

where $k_{B}$is the Boltzmann constant, $T$is the temperature, $\nabla T$ is the gradient of temperature, $e$ is the electron charge, ${S_{T}}_{2}$ is the Soret coefficient of the Cl^-^ ion, $S_{T_{1}}$is the Soret coefficient of the CTAC micelle, $N_{\mathrm{agg}}$ is the number of CTA^+^ ions in a micelle (assumed to be 89) and $Z_{1}$ is the effective charge on the micelle, $S_{C}$ is the Seebeck coefficient resulting from the Soret coefficients of individual charged species. The thermo-electric field ($E_{T})$is then decomposed into tangential and normal components of the surface and the net thermo-electric force ($F_{E_{T}})$ is numerically integrated as

|  | $F_{E_{T}}= \int_{A} \sigma E_{T,\parallel}dA$ | (2) |
| --- | --- | --- |

where $\sigma$ is the surface charge density, and $E_{T,\parallel}$ is the tangential component of thermo-electric field. The surface charge density of the Janus particle is assumed to be a constant and is given as a function of zeta potential

|  | $\sigma=\frac{\epsilon k_{B}T}{e\lambda}\left[ \exp\left( \frac{e\zeta}{2k_{B}T} \right)-\exp\left( -\frac{e\zeta}{2k_{B}T} \right)-\frac{4\lambda}{a}\frac{\left( \exp\left( \frac{e\zeta}{2k_{B}T} \right)-1 \right)}{\left( \exp\left( \frac{e\zeta}{2k_{B}T} \right)+1 \right)} \right]$ | (3) |
| --- | --- | --- |

where $\epsilon$ is the dielectric permittivity of the particle, $\lambda$ is the Debye length which is measured as a function of concentration of CTAC and $\zeta$ is the measured zeta potential of the particle.

Because of the asymmetry of the Janus particle, the difference between the net thermoelectric force on each of the two halves causes a torque that rotates the particle. The net thermoelectric torque is given as

|  | $T_{F_{E,T}}= \oint\bar{r}\times\sigma E_{T,\parallel}dA$ | (4) |
| --- | --- | --- |

where $\bar{r}$ is the position vector relative to the center of the particle.

It should be noted that, the depletion force can be neglected because of the low concentration of CTAC. More discussions on concentration-dependent thermoelectricity are addressed in our previous work. ^1,2^

**Note S2: Off-axis rotation driven by the thermoelectric force and optical force**


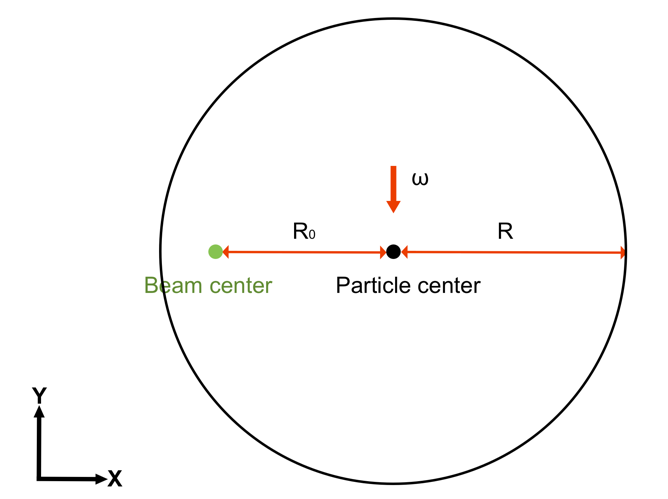


**Fig. S10:** Torque calculation of the in-plane rotation around the laser beam.

For the rotation of Janus PS/Au particles, the thermoelectric torque $F_{\mathrm{tY}}R_{0}$ will balance with the optical torque $F_{\mathrm{oY}}R_{0}$and the Stokes drag torque $F_{d}R_{0}$ in the Z-axis, *i.e*., $F_{\mathrm{tY}}R_{0}-F_{\mathrm{oY}}R_{0}{=F}_{d}R_{0}$, to maintain a stable rotational rate around Z-axis (Fig. S10). The Stokes drag force $F_{d}$ is caused by both the translation of the center of mass and the spinning relative the center of mass,

|  | $F_{d}R_{0}=L_{s}+L_{\mathrm{center}}$ | (5) |
| --- | --- | --- |

where *L*_S_ is the torque of spinning, $\frac{L_{s}}{8\pi\eta R^{3}\omega}=\zeta\left( 3 \right)-3(\frac{\pi^{2}}{6}-1)\frac{h}{R}$ ^3^, while $\frac{L_{\mathrm{center}}}{6\pi\eta RR_{0}^{2}\omega}=f=(1-\frac{9R}{16\left( R+h \right)}+\frac{R^{3}}{8\left( R+h \right)^{3}}-\frac{45R^{4}}{256\left( R+h \right)^{4}}-$ $\frac{R^{5}}{16\left( R+h \right)^{5}}){}^{-1}$ ^4^ (*h* is the non-zero gap distance between the particle and the substrate, which is estimated as 30 nm considering the balance between the electrostatic repulsion force and the van der Waals force ^1^. *R* is the radius of the particle). It’s worth noting that the substrate effect has already been included in the derivations of *L*_s_ and *L*_center_.

In the X axis, the thermoelectric force $F_{\mathrm{tX}}$will overcome the optical force $F_{\mathrm{oX}}$and a static friction force *F*_r_ (See Fig.S7 caption), providing the centripetal force for circular motion,

|  | $F_{\mathrm{tX}}$-$F_{\mathrm{oX}}$=*F*_r_ + $m\omega^{2}R_{0}$ | (6) |
| --- | --- | --- |

**Note S3: Algorithms for feedback control method**

Providing a Janus particle is aimed to a target located at **P**_t_, our program will execute the following steps at each frame of the CCD camera:

i) Grab three instantaneous positions: (1) the geometrical center of the Janus paritcle, ***P***_sc_(*t*); (2) the brightest point of the Janus particle, ***P***_sb_(*t*); (3) the geometrical center of the target, ***P***_t_(*t*).

ii) The orientation of the Janus particle can be determined by ***P***_sb_(*t*) - ***P***_sc_(*t*), *i.e* from the center of mass to the brightest point. The program calculates the orientation *θ*(*t*), and the target angle between the Janus particle and the target *α*(*t*):

$$\theta\left( t \right)=\left\{ \begin{aligned} 2\pi-arccos\{\frac{X\left[ \boldsymbol{P}_{\mathrm{sb}}\left( t \right) \right]-X\left[ \boldsymbol{P}_{\mathrm{sc}}\left( t \right) \right]}{\sqrt{{{[\boldsymbol{P}}_{\mathrm{sb}}\left( t \right)-\boldsymbol{P}_{\mathrm{sc}}\left( t \right)]}^{2}}}\}, &Y\left[ \boldsymbol{P}_{\mathrm{sc}}\left( t \right) \right]-Y\left[ \boldsymbol{P}_{\mathrm{sc}}\left( t \right) \right]<0 \\ arccos\{\frac{X\left[ \boldsymbol{P}_{\mathrm{sb}}\left( t \right) \right]-X\left[ \boldsymbol{P}_{\mathrm{sc}}\left( t \right) \right]}{\sqrt{{{[\boldsymbol{P}}_{\mathrm{sb}}\left( t \right)-\boldsymbol{P}_{\mathrm{sc}}\left( t \right)]}^{2}}}\}, &Y\left[ \boldsymbol{P}_{\mathrm{sc}}\left( t \right) \right]-Y\left[ \boldsymbol{P}_{\mathrm{sc}}\left( t \right) \right]\geq0 \end{aligned} \right.$$

$$\alpha\left( t \right)=\left\{ \begin{aligned} 2\pi-arccos\{\frac{X\left[ \boldsymbol{P}_{t}\left( t \right) \right]-X\left[ \boldsymbol{P}_{\mathrm{sc}}\left( t \right) \right]}{\sqrt{{{[\boldsymbol{P}}_{t}\left( t \right)-\boldsymbol{P}_{\mathrm{sc}}\left( t \right)]}^{2}}}\}, &Y\left[ \boldsymbol{P}_{t}\left( t \right) \right]-Y\left[ \boldsymbol{P}_{\mathrm{sc}}\left( t \right) \right]<0 \\ arccos\{\frac{X\left[ \boldsymbol{P}_{t}\left( t \right) \right]-X\left[ \boldsymbol{P}_{\mathrm{sc}}\left( t \right) \right]}{\sqrt{{{[\boldsymbol{P}}_{t}\left( t \right)-\boldsymbol{P}_{\mathrm{sc}}\left( t \right)]}^{2}}}\}, &Y\left[ \boldsymbol{P}_{t}\left( t \right) \right]-Y\left[ \boldsymbol{P}_{\mathrm{sc}}\left( t \right) \right]\geq0 \end{aligned} \right.$$

iii) Two conditions are considered.

Condition 1 – the Janus particle is rotating: Compare *θ*(*t*) with the angle range [*α*(*t*)-*β*, *α*(*t*)+*β*] (*β* is the tolerance value input). If *α*(*t*)-*β*≤ *θ*(*t*) ≤*α*(*t*)+*β*, the particle will be switched to the swimming state. Otherwise, the particle keeps in the rotation state.

Condition 2 – the Janus particle is swimming: If *α*(*t*)-*β*≤ *θ*(*t*) ≤*α*(*t*)+*β*, the particle stays in the swimming state. Otherwise, all the laser beams will be switched off and the particle will be aligned with the center of the laser beams by either moving the stage or moving the laser beams. Then, the rotation state of the particle will be activated.

iv) Repeat the above steps in sequence.

We have tested the accuracy of the swimming direction of a 5 μm PS/Au Janus particle during 0-360° with an interval of 30°. The angle *α* is assigned with twelve values (*α* = 15°, 45°, 75°……345°) and the tolerance angle *β* is set as 15°.

**References:**

1. Lin, L.H. et al., Opto-thermophoretic assembly of colloidal matter. *Science Advances* **3**, e1700458 (2017).

2. Lin, L.H. et al. Opto-thermoelectric nanotweezers. *Nature Photonics* **12**, 195-201 (2018).

3. Liu, Q. & Prosperetti, A. Wall effects on a rotating sphere. Journal of Fluid Mechanics **657**, 1-21, (2010).

4. Ambari, A., Manuel, B. G. & Guyon, E. Effect of a plane wall on a sphere moving parallel to it. Journal de Physique Lettres **44**, 143-146 (1983).
